# Randomized Clinical Trial for Early Postoperative Complications of Ex-PRESS Implantation versus Trabeculectomy: Complications Postoperatively of Ex-PRESS versus Trabeculectomy Study (CPETS)

**DOI:** 10.1038/srep26080

**Published:** 2016-05-17

**Authors:** Shogo Arimura, Yuji Takihara, Seiji Miyake, Kentaro Iwasaki, Makoto Gozawa, Takehiro Matsumura, Takeshi Tomomatsu, Yoshihiro Takamura, Masaru Inatani

**Affiliations:** 1 Department of Ophthalmology, Faculty of Medical Sciences, University of Fukui, 23-3 Shimoaizuki, Matsuoka, Eiheiji, Yoshida, Fukui, 910-1193, Japan

## Abstract

We compared early postoperative complications between trabeculectomy and Ex-PRESS implantation. Enrolled patients with 39 primary open-angle or 25 exfoliative glaucoma were randomly assigned to receive trabeculectomy (trabeculectomy group) or Ex-PRESS implantation (Ex-PRESS group). Primary outcomes were early postoperative complications, including postoperative anterior chamber inflammation, frequencies of hyphema, flat anterior chamber, choroidal detachment, hypotonic maculopathy, and the change of visual acuity. The postoperative flare values in trabeculectomy group were higher than those in the Ex-PRESS group (overall, *P* = 0.004; and 10 days, *P* = 0.02). Hyphema occurred significantly more frequently in the trabeculectomy group (*P* = 0.0025). There were no significant differences of the other primary outcomes between the two groups. Additionally, duration of anterior chamber opening was significantly shorter in the Ex-PRESS group (*P* = 0.0002) and the eyes that had iris contact with Ex-PRESS tube had significantly shallower anterior chambers than did the eyes without the iris contact (*P* = 0.013). The Ex-PRESS implantation prevented early postoperative inflammation and hyphema in the anterior chamber and shortened the duration of anterior chamber opening. Iris contact with the Ex-PRESS tube occurred more frequently in eyes with open-angle glaucoma and shallow anterior chambers.

Trabeculectomy is the gold-standard filtering surgery for intraocular pressure (IOP) reduction in glaucoma patients^1^. In this procedure, the surgeon creates a scleral flap for the drainage of the aqueous humor and subsequent bleb formation. The rates of success and specific complications with trabeculectomy are widely known^2^,^3^. Short-term complications include a flat anterior chamber, hypotony, hyphema, and choroidal detachment. Long-term complications include bleb leaks, blebitis, and bleb failure. These complications frequently deteriorate the quality of vision postoperatively.

The Ex-PRESS glaucoma filtration device (Alcon Laboratories, Fort Worth, TX, USA) is a miniature, non-valved drainage tube that has been used as an alternative to trabeculectomy; this device shunts aqueous humor from the anterior chamber into the subconjunctival area near the limbus. Ex-PRESS is designed for the stable filtration of the aqueous humor although its flow (0.09 mmHg outflow resistance at physiologic flow rates) needs to be regulated by the suture tension of scleral flap^4^. In addition, no conventional sclerectomy or iridectomy is required for this procedure. Therefore, Ex-PRESS implantation is expected to reduce the number of postoperative complications after filtering surgery.

There have been several prospective and retrospective studies that have compared the safety and efficacy of Ex-PRESS implantation with those of trabeculectomy^5^,^6^,^7^,^8^,^9^,^10^,^11^,^12^. Two meta-analyses that included four previous prospective studies have illustrated that Ex-PRESS implantation provided IOP reduction comparable to that provided by trabeculectomy; additionally, they caused fewer anterior chamber hyphemas than those caused by trabeculectomy^13^,^14^.

The objective of this study, called “Complications Postoperatively of Ex-PRESS versus Trabeculectomy Study (CPETS),” was to compare early postoperative complications between trabeculectomy and Ex-PRESS implantation in Asian eyes that had medically-uncontrolled primary open-angle glaucoma (primary OAG) or exfoliative glaucoma. Our primary outcome measure included postoperative anterior chamber inflammation because this surgical procedure appeared to induce less inflammation than trabeculectomy. Moreover, there have been case reports about the effects of iris contact with the tube^15^,^16^. Despite the shallower anterior chambers in Asian eyes compared with Caucasian eyes, the frequency of iris contact and its relationship with anterior chamber depth still remains unknown. Our second outcome measure included the frequency of iris contact after Ex-PRESS implantation.

## Methods

### Patient selection

CPETS was approved by the institutional review board of Fukui University Hospital, Fukui, Japan. This study was registered with the University Hospital Medical Information Network Clinical Trials Registry of Japan (identifier University Hospital Medical Information Network 000008680; date of access and registration, August 15, 2012). The protocol adhered to the tenets of the Declaration of Helsinki. Written informed consent was obtained from all subjects. Patients were recruited between August 30, 2012 and February 13, 2015 at Fukui University Hospital. The enrolled patients were randomly assigned to receive either a trabeculectomy or an Ex-PRESS implantation. Allocation was concealed until intervention was assigned. Yo. T. generated the random allocation sequence, M.I. and Yu T. enrolled participants and S.M. assigned participants to interventions. The enrolled patients were determined with the following criteria: (1) Japanese patients with primary OAG or exfoliative glaucoma (The presence of OAG was diagnosed by open-angle through gonioscopic examinaton, glaucomatous optic nerve head appearance with a cup/disk ratio of 0.7 or larger, and glaucomatous visual field defect at least satisfying the criteria proposed by Anderson and Patella.), (2) minimum age of 20 years, (3) no previous history of ocular surgery, and (4) an IOP ≥18 mmHg (despite maximum tolerated administration of anti-glaucoma medication). The exclusion criteria were as follows: (1) eyes that had not been treated with anti-glaucoma medication before surgery, (2) had a history of uveitis, or (3) were allergic to metals. For each patient, if both eyes satisfied the inclusion criteria, the eye with the higher IOP was selected for the study. The preoperative IOP was the average of the measurements taken within two months prior to the operation.

### Surgical procedures

All trabeculectomy and Ex-PRESS implantation procedures were performed using identical processes during the study period. A 5-mm conjunctival incision was created along the limbus to construct a fornix-based conjunctival flap, and a 4-mm wide half-layer scleral flap was formed. Sponges (M.Q.A., Inami, Tokyo, Japan) soaked in 0.04% mitomycin-C (0.4 mg/mL) was applied on and under the scleral flap and under the conjunctiva for 4 min, followed by irrigation with 200 mL of physiological saline. In the eyes that received a trabeculectomy, a deep block of limbal tissue was excised to create a fistula into the anterior chamber, and a peripheral iridectomy was performed. In the eyes that received an Ex-PRESS implantation, the tube was inserted into the anterior chamber after penetration with a 25-gauge needle. In both procedures, the scleral flap and conjunctiva were then sutured with 10-0 nylon. All patients received similar postoperative topical medications: 0.5% levofloxacin for 3 weeks and 0.1% betamethasone three times daily for 6 months without tapering after operation respectively. Laser suture lysis and bleb needling were completed within 1 month of surgery, depending on the postoperative IOP and formation of the bleb.

### Primary outcome measures

The primary outcomes for this study were the early postoperative complications, including postoperative anterior chamber inflammation, the frequencies of hyphema, a flat anterior chamber, choroidal detachment, hypotonic maculopathy, and visual acuity. The inflammation in the anterior chamber was quantified using a flare meter (FM-600, Kowa, Tokyo, Japan). Hyphema was defined as blood deposition in the anterior chamber as assessed during the postoperative slit lamp examination. A flat anterior chamber was identified if the anterior chamber at the pupillary border of the iris was narrower than the corneal thickness or if cornea–iris contact was observed at the peripheral anterior chamber. Choroidal detachment was determined during the postoperative fundus examination. Hypotonic maculopathy was defined^17^ as IOP below 6 mmHg, retinal folds in the macula and decreased visual acuity. Visual acuity was measured as best corrected visual acuity. A logarithm of the reciprocal of the decimal BCVA was used to approximate the logarithm of minimal angle of resolution.

### Secondary outcome measures

The secondary outcomes were the duration of the anterior chamber opening during surgery, postoperative IOP, postoperative anti-glaucoma medications, laser suture lysis, bleb needling, and the relationship between the iris contact of the Ex-PRESS tube and the anterior chamber depth in the Ex-PRESS group. The time for which the anterior chamber was open during the surgery was defined as the time between starting the incision for the fistula creation in the anterior chamber and suturing the scleral flap in the trabeculectomy group. The same was defined as the time between the penetration with a 25-gauge needle and suturing the scleral flap in the Ex-PRESS group. The postoperative IOPs were measured with Goldmann applanation tonometry. Because the anterior chamber depth changed with hypotony after the surgery, it was measured preoperatively using an optical biometer (OA-1000, TOMEY, Aichi, Japan). We performed bleb revision with needling if the postoperative eyes had higher IOP than preoperative IOP despite of the completion of laser suture lysis. Needling of the bleb was performed by sweeping to cut episcleral scarring that was obstructing the intrascleral pathway. The contact of the iris with the Ex-PRESS tube was judged during a slit lamp examination at 1 month or later when there was no shallow or flat anterior chamber due to hypotony present. The anterior chamber angle examination was performed by gonioscopy before surgery to exclude angle closure glaucoma and again after the surgery to examine the position of the insertion of the Ex-PRESS device in the angle.

### Data collection of patient characteristics

The other data regarding patient characteristics were gender, age, glaucoma type, and other preoperative ophthalmic data, including preoperative IOP, number of medications, corneal thickness, anterior chamber depth, axial length, best-corrected visual acuity, and visual field. Corneal thickness, anterior chamber depth, and axial length were measured with optical coherence interferometry (OA-1000; Tomey, Aichi, Japan). A logarithm of the reciprocal of the decimal BCVA was used to approximate the logarithm of the minimal angle of resolution (LogMAR). Visual field testing was performed using a static automated white-on-white threshold 24-2 perimetry program, SITA Standard (Model 750; Zeiss, Tokyo, Japan). If the eye had advanced visual field loss, a 10-2 perimetry program was used.

### Follow-up visit

CPETS was planned to examine postoperative complications for five years. To determine the early postoperative complications in the study, we examined the patients at days 1, 3, 7, and 10, and also at 1 and 3 months after the surgery. If the patients required additional treatment for complications, they were examined at additional follow-up visits.

### Sample size

The sample size which would provide 80% power to prove (at a one-sided a level of 0.05) the superiority of Ex-PRESS implantation compared with trabeculectomy for postoperative anterior chamber inflammation was 28 eyes in each group for an effect size of 0.7. Therefore, we had planned to study 64 eyes to account for a 12% dropout rate.

### Statistical analysis

The univariate analysis was performed with the Mann-Whitney nonparametric test (with Bonferroni correction) and the Chi-square test. *P* values of <0.05 were considered statistically significant. The data were analyzed with the JMP statistical package, version 10.0 (SAS Institute, Inc. Cary, NC, USA).

## Results

### Patient recruitment

**Figure 1:**
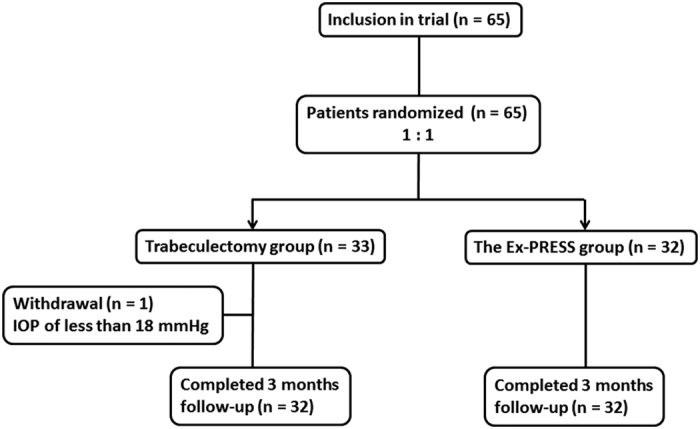
Diagram of the study design. Patients who met the inclusion criteria were randomized 1:1 to receive a trabeculectomy or Ex-PRESS implantation. After randomization, one patient in the trabeculectomy group was excluded because the preoperative intraocular pressure (IOP) was less than 18 mmHg.

In total, 65 patients were recruited to this study. One patient whose eye had a preoperative IOP of less than 18 mmHg did not meet the criteria. Thirty-two patients were assigned to each arm. All patients completed the follow-up visits for the early postoperative complication evaluations (Fig. 1).

### Demographic characteristics

There were no significant differences between the trabeculectomy and the Ex-PRESS groups for the baseline characteristics, such as age, gender, the side of the operated eye (right or left), type of glaucoma (primary OAG or exfoliative), the number of preoperative medications, preoperative IOP, visual acuity (logMAR), visual field defect, corneal thickness, anterior chamber depth, or axial length (Table 1).

### Primary outcome measures

**Figure 2:**
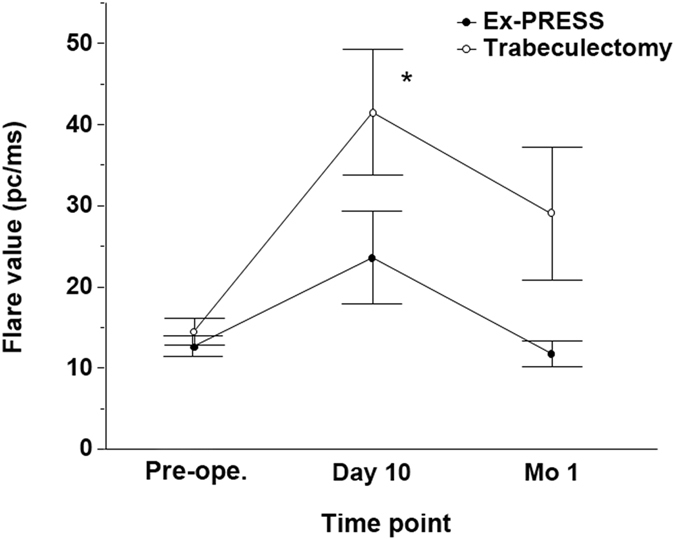
Changes in flare value after a trabeculectomy or Ex-PRESS implantation. There were significant differences between the groups 10 days after the surgery (*P = 0.04). The data (mean ± standard error) were compared with the Mann-Whitney test with Bonferroni correction. Pre-ope.; pre-operation.

Figure 2 shows the results of the changes of inflammation as quantified with a flare meter between the trabeculectomy group and Ex-PRESS group. First, to test whether the overall flare value difference between the trabeculectomy and Ex-PRESS groups was statistically significant, the values which exceeded the mean +2 standard deviation of preoperative flare values were defined as high flare value (more than 29.97 pc/ms). The numbers of eyes with high flare values within 1 month after the surgery were compared between the trabeculectomy and Ex-PRESS groups. The numbers of eyes with high flare values were 16 eyes in the trabeculectomy group and 5 eyes in the Ex-PRESS group (overall, *P* = 0.004, the Chi-square test).

Next, the statistically significant difference of each flare value at each time point between the trabeculectomy and Ex-PRESS groups was evaluated. The numbers of eyes with high flare values were 15 eyes versus 5 eyes 10 days after the surgery (*P* = 0.02, the Chi-square test with Bonferroni correction); 6 eyes versus 1 eyes 1 month after the surgery (*P* = 0.10, the Chi-square test with Bonferroni correction), in the trabeculectomy group versus the Ex-PRESS group, respectively. The trabeculectomy group also showed significantly higher flare values than the Ex-PRESS group 10 days after the surgery (*P* = 0.04, the Mann-Whitney test with Bonferroni correction).

Hyphema occurred in 8 (25%) of 32 eyes in the trabeculectomy group, while no eyes (0%) in the Ex-PRESS group developed a hyphema (*P* = 0.0025). There were no significant differences between the two groups for flat anterior chamber (*P* = 0.45), choroidal detachment (*P* = 0.49), and hypotonic maculopathy (*P* = 1.00) (Table 2). Postoperative hypotony occurred in 9 of 32 (28.1%) in both the groups.

There was no significant difference in preoperative visual acuity between the two groups as represented by logMAR values (0.33 ± 0.48 in the trabeculectomy group vs. 0.40 ± 0.43 in the Ex-PRESS group; *P* = 0.28). The postoperative visual acuity also did not show any significant differences between the two groups (0.44 ± 0.48 vs. 0.53 ± 0.40; *P* = 0.12 at 1 month, 0.41 ± 0.47 vs. 0.56 ± 0.48; *P* = 0.076 at 3 months in the trabeculectomy group vs. the Ex-PRESS group, respectively).

### Secondary outcome measures

The mean ± SD duration for an open anterior chamber during surgery was 4.9 ± 1.0 min in the trabeculectomy group and 4.1 ± 1.0 min in the Ex-PRESS group. The duration was significantly shorter in the Ex-PRESS group (*P* = 0.0002).

The postoperative IOPs are shown in Table 3. There were no significant differences in the postoperative IOPs or in the preoperative IOPs between the two groups at any follow-up visits. No eyes were treated with anti-glaucoma medication during the three months of postoperative evaluations. Ocular massage was performed in 23 of 32 (71.9%) in the trabeculectomy group and in 19 of 32 (59.4%) in the Ex-PRESS group. Laser suture lysis was performed in 20 (62.5%) of 32 eyes in the trabeculectomy group and in 24 (75.0%) of 32 eyes in the Ex-PRESS group (*P* = 0.28). Bleb revision with needling was performed in 3 (10%) of 32 eyes in the trabeculectomy group and in 3 (10%) of 32 eyes in the Ex-PRESS group (*P* = 1.00).

### The subgroup analysis for the relationship between iris contact with the Ex-PRESS tube and anterior chamber depth

**Figure 3:**
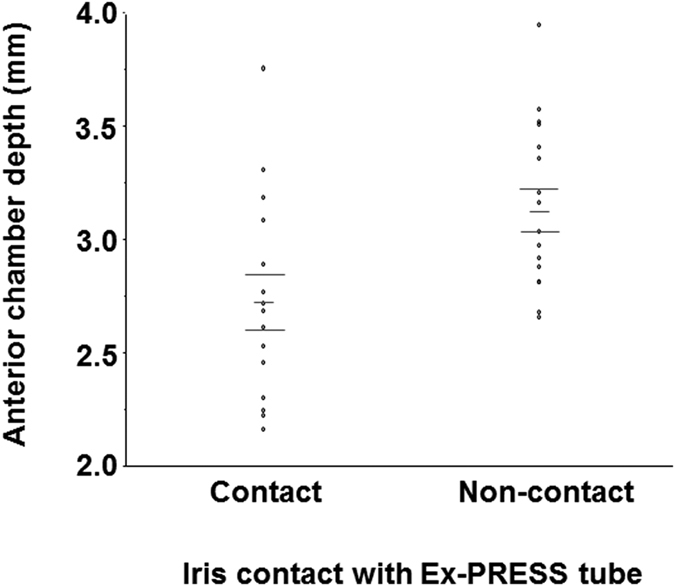
The difference in the anterior chamber depth in eyes with or without iris contact with the Ex-PRESS tube. The data (mean ± standard deviation) were compared with the Mann-Whitney non-parametric test.

Fifteen (46.9%) of 32 eyes in the Ex-PRESS group had iris contact with the Ex-PRESS tube after the surgery (Fig. 3). The eyes that had iris contact had significantly shallower anterior chambers than did the eyes that did not have the iris contact (2.7 ± 0.1 mm in eyes with iris contact versus 3.1 ± 0.4 mm in eyes without iris contact; *P* = 0.013). All seven eyes with a preoperative anterior chamber depth of ≤2.61 mm developed iris contact with the Ex-PRESS tube.

## Discussion

The main aim of our present study was the comparison of the frequency of early postoperative complications between trabeculectomy and Ex-PRESS implantation. The inflammation in the anterior chamber was significantly lower in the Ex-PRESS group after the surgery. The frequency of postoperative hyphema was also lower in the Ex-PRESS group than in the trabeculectomy group. The duration of the open anterior chamber during surgery was also significantly shorter in the Ex-PRESS group. Additionally, in the Ex-PRESS group, 46.9% eyes had iris contact with the Ex-PRESS tube, which was significantly associated with shallower anterior chambers before surgery.

No conventional sclerectomy or iridectomy is required for the Ex-PRESS implantation. The surgery, which is categorized as a minimally invasive glaucoma surgery procedure, seems to induce less inflammation than trabeculectomy. Stable filtration after Ex-PRESS implantation may be expected to show less IOP fluctuation during the intraoperative and early postoperative periods. Hypotony due to overfiltration increases the vascular permeability of the iris and the protein effusion from the choroidal detachment. Our hypothesis is that Ex-PRESS implantation presents with a lower chance of postoperative inflammation than trabeculectomy. There have been several retrospective^9^,^11^,^12^,^18^ and four randomized clinical trials^6^,^7^,^8^,^19^ that have compared these methods. These studies indicated that IOP reduction after Ex-PRESS implantation was comparable to that in trabeculectomy but there were fewer postoperative complications with Ex-PRESS implantation than with trabeculectomy. Meta-analyses of the four randomized controlled trials^13^,^14^ showed that the IOP reduction efficiency and the frequency of the complications except for hyphema were comparable between the two procedures. Therefore, the question of invasive levels for Ex-PRESS implantation compared with trabeculectomy is still controversial. Our randomized clinical trial was unique because the lower level of flare values with Ex-PRESS implantation has been shown by the quantification with flare meter.

The Ex-PRESS implantation, in addition to less postoperative inflammation, also presented with a significantly shorter open anterior chamber duration (*P* = 0.0002) and a lower frequency of hyphema (*P* = 0.0025) than did trabeculectomy. Instead of penetration into the anterior chamber with a 25-gauge needle and Ex-PRESS tube insertion, a limbal block incision and iridectomy are performed during a trabeculectomy. The 0.8 min of time difference between the two groups implies that Ex-PRESS tube insertion was simpler to perform than trabeculectomy. Intraoperative hypotony occurs during a limbal block incision in trabeculectomy and penetration into the anterior chamber with a 25-gauge needle in the Ex-PRESS implantation, leading to the increased risk for surgical complications, such as expulsive hemorrhage, corneal decompensation, and postoperative inflammation in the anterior chamber. Shorter anterior chamber opening in the Ex-PRESS implantation might be preferable to reduce these complications. The shortened duration for an open anterior chamber offered by the Ex-PRESS implantation seems small although the difference of the duration was significant (*P* = 0.0002). Because all the surgeries were performed by two glaucoma specialists (M.I & Yu T), the time difference between the 2 procedures might have been small. The time difference between the 2 procedures might have been larger if other surgeons carried out the study.

Most previous studies have reported a lower frequency of hyphema after Ex-PRESS implantation^13^,^14^,^18^, which is consistent with our present results exhibiting 8 of 32 eyes in the trabeculectomy group versus 0 of 32 eyes in the Ex-PRESS group. Hyphema is the most common early postoperative complication after trabeculectomy^20^,^21^ with a reported incidence of 3.9% to 56%^20^,^22^,^23^,^24^. Although most hyphemas are transient, they can cause visual disturbances during the early postoperative period. Most intraoperative bleeding in the anterior chamber occurs after the iridectomy procedure, which may explain the absence of hyphema in eyes with Ex-PRESS implantation. Some randomized clinical trials about trabeculectomy showed less frequent incidence of hyphema compared to the trabeculectomy group in the present study did^20^,^25^. The incidence of hyphema seems to depend on the definition of hyphema, the study designs which are either prospective or retrospective, and the primary outcome about either complications or efficacy. The high frequency may be because we counted hyphema if blood deposition in the anterior chamber was found at least once during follow-up examination in a prospective manner focusing surgical complications.

Less deterioration of visual acuity, more rapid visual recovery, and fewer postoperative visits with Ex-PRESS implantation have been reported in three studies^8^,^12^,^18^. Less inflammation by the surgery apparently leads to better vision after the surgery. However, we could not find any significant differences in postoperative visual acuity between the two groups. Our study did not have inclusion criteria for preoperative visual acuity, which included various visual acuity and visual field losses among the patient characteristics. The variance in the baseline data may have resulted in no difference in postoperative vision between the two groups.

Fifteen of the 32 eyes had iris contact with the Ex-PRESS tube. There have been a few case reports^15^,^16^ about iris contact with the Ex-PRESS tube. Because Ex-PRESS implantation has been performed in both European countries and the United States, the iris contact may have been a rare complication. The anterior chambers of Asian eyes, including Japanese eyes, are shallower than those of European and African descendents^20^. Moreover, the present study only recruited phakic eyes. High frequency of iris contact in the present study may be due to the inclusion criteria for Asian phakic eyes. One case of iris contact required an additional surgery for the removal of the Ex-PRESS tube because of ocular pain^26^. Because all the eyes with an anterior chamber depth of ≤2.61 mm developed iris contact with the Ex-PRESS tube, trabeculectomy or a combined procedure with Ex-PRESS implantation and lens extraction may be favored for Asian OAG eyes with shallow anterior chambers.

Our study had some limitations. The study was an open-label trial similar to the previous four trials. The lack of masking to the procedure added a possibility of bias to this study. The present data only showed early postoperative complications; however, we have not determined the long-term postoperative complications. CPETS will examine long-term postoperative complications, such as the reduction of corneal endothelial cells, cataract progression, bleb leak, bleb infection, and re-operation due to bleb fibrosis. Long-term follow-up will reveal any further differences in complications between the two procedures.

In our conclusion, the Ex-PRESS implantation can prevent early postoperative inflammation and anterior chamber hyphema and can shorten the duration of anterior chamber opening compared to trabeculectomy. Other early complications and postoperative visual acuity findings were comparable between the two procedures. Iris contact with the Ex-PRESS tube frequently occurred in Asian phakic eyes with OAG and shallower anterior chambers.

## Additional Information

**How to cite this article**: Arimura, S. *et al*. Randomized Clinical Trial for Early Postoperative Complications of Ex-PRESS Implantation versus Trabeculectomy: Complications Postoperatively of Ex-PRESS versus Trabeculectomy Study (CPETS). *Sci. Rep.* **6**, 26080; doi: 10.1038/srep26080 (2016).

## Figures and Tables

**Table 1:** Demographic characteristics.

Characteristic	Trabeculectomy	Ex-PRESS	*P* value
	n = 32	n = 32	
Age, y	72.7 ± 9.6	70.7 ± 11.4	0.47*
Sex, Male/Female	13/19	14/18	0.80^†^
Side of surgical eye, Right/Left	17/15	14/18	0.45^†^
Type			
Primary open-angle glaucoma/Exfoliation glaucoma	19/13	20/12	0.80^†^
Number of preoperative medication	2.7 ± 0.7	2.6 ± 0.7	0.27*
Anterior chamber depth, mm	2.93 ± 0.45	2.94 ± 0.45	0.94*
Preoperative intraocular pressure, mmHg	27.9 ± 10.7	27.2 ± 8.5	0.94*
Preoperative visual acuity, logMAR	0.33 ± 0.48	0.40 ± 0.43	0.28*
Axial length, mm	23.6 ± 1.6	24.2 ± 1.5	0.13*
Corneal thickness, mm	0.51 ± 0.05	0.51 ± 0.04	0.95*
Automated perimetry program 24-2			
Eyes, No. (%)	11 (34)	16 (50)	
MD, mean (dB)	−10.2 ± 6.94	−11.3 ± 5.52	0.75*
Automated perimetry program 10-2			
Eyes, No. (%)	18 (56)	13 (41)	
MD, mean (dB)	−22.0 ± 7.60	−22.0 ± 8.96	0.81*
Fixation loss			
No. (%)	3 (9)	3 (9)	1.00

logMAR = logarithm of the minimal angle of resolution, MD = mean deviation.

*Mann-Whitney non-parametric test. (Data are mean values ± standard deviation).

^†^The Chi-square test.

**Table 2:** Early postoperative complications.

Type of complication	Trabeculectomy n = 32	Ex-PRESS n = 32	*P* value
Hyphema (%)	8 (25.0%)	0 (0%)	0.0025*
Flat anterior chamber (%)	5 (15.6%)	3 (9.4%)	0.45
Choroidal detachment (%)	4 (12.5%)	6 (18.8%)	0.49
Hypotonic maculopathy (%)	1 (3.1%)	1 (3.1%)	1.00

The Chi-square test test.

*Statistically significant (*P* < 0.05).

**Table 3:** Intraocular pressure in the trabeculectomy and the Ex-PRESS groups.

Time Point	Trabeculectomy Intraocular pressure (mmHg) n = 32	Ex-PRESS Intraocular pressure (mmHg) n = 32	*P* value
Preoperatively	27.9 ± 10.7	27.2 ± 8.5	0.94
1 day	12.2 ± 6.1	10.4 ± 7.9	0.15
3 day	10.8 ± 5.1	9.4 ± 5.9	0.28
7 day	8.8 ± 3.4	7.5 ± 3.3	0.11
10 day	9.5 ± 3.4	8.3 ± 3.3	0.08
1 month	13.3 ± 4.8	15.8 ± 7.7	0.40
3 month	13.4 ± 3.8	14.7 ± 6.0	0.48

Mann-Whitney non-parametric test (Data are mean values ± standard deviation).
